# Real-World Experience with Approved CAR T-Cell Therapies Ciltacabtagene Autoleucel and Idecabtagene Vicleucel in 1272 Relapsed/Refractory Multiple Myeloma Patients

**DOI:** 10.3390/cancers18061013

**Published:** 2026-03-20

**Authors:** Charalampos Filippatos, Ioannis Ntanasis-Stathopoulos, Alexandros Briasoulis, Panagiotis Malandrakis, Evangelos Terpos, Maria Gavriatopoulou

**Affiliations:** Department of Clinical Therapeutics, School of Medicine, National and Kapodistrian University of Athens, 11528 Athens, Greece

**Keywords:** CAR T, ide-cel, cilta-cel, real-world, retrospective, myeloma

## Abstract

**Background:** Ciltacabtagene autoleucel (cilta-cel) and idecabtagene vicleucel (ide-cel) have transformed the treatment landscape of relapsed/refractory multiple myeloma (RRMM). Given their recent regulatory approval and limited availability, mainly due to logistical issues, real-world data remain scarce. **Methods:** A retrospective study was conducted using the TriNetX database, identifying adult patients with RRMM treated with either cilta-cel or ide-cel. The clinical outcomes evaluated included overall survival (OS), progression-free survival (PFS), as well as the safety profile. **Results:** A total of 697 patients treated with cilta-cel and 575 with ide-cel were identified. The median age was 65 and 67 years, with ~16% being Black/African American. The 12-month OS was 89.6% for cilta-cel and 86.0% for ide-cel. In a descriptive subgroup analysis, renal impairment (eGFR < 60 mL/min/1.73 m^2^) seemed to be associated with significantly inferior OS in both cohorts (HR = 3.66, *p* < 0.001 for cilta-cel; HR = 1.73, *p* = 0.003 for ide-cel). Conversely, prior anti-CD38 exposure did not seem to impact survival in any of the two treatment groups. Any-grade CRS occurred in 45.9% (cilta-cel) and 41.8% (ide-cel), while any-grade ICANS was observed in 15.4% and 11.8%, respectively. Severe (grade ≥ 3) ICANS remained rare (<3%) in both cohorts. Hematologic toxicity was prevalent, with grade ≥ 3 neutropenia occurring in 76.0% (cilta-cel) and 68.0% (ide-cel). Notably, any-grade infections (28.5–40.1%) and hypogammaglobulinemia (41.1–43.1%) were frequent, highlighting a significant long-term immunosuppressive burden. **Conclusions:** In these real-world cohorts, both approved CAR T-cell therapies demonstrated favorable survival outcomes. While the incidence of severe hematologic and immune-related toxicities was high, these findings are compatible with published data from clinical trials and it seems that the clinical utility of these drugs overcomes the adverse safety profile.

## Simple Summary

Multiple myeloma is a type of blood cancer that remains difficult to treat when it returns after treatment. Recently, advanced therapies called CAR T-cells have gained approval, but data on their use in everyday clinical practice is limited. This study analyzed real-world data from 1272 patients treated with the two approved CAR T-cell products (cilta-cel and ide-cel). The one-year survival rates were approximately 90% for cilta-cel and 75% for ide-cel. Notably, prior exposure to anti-CD38 antibodies—now a standard in the frontline setting—did not significantly impact survival outcomes. Conversely, baseline renal impairment was associated with significantly inferior survival. While any-grade CRS and ICANS were observed in approximately 42–46% and 12–15% of patients, respectively, severe events remained rare (<3%). High rates of severe low blood counts and infections were prevalent, highlighting a significant immunosuppressive burden. These findings confirm that CAR T-cell therapies are effective and feasible in diverse real-world populations.

## 1. Introduction

Multiple myeloma (MM) is a plasma cell dyscrasia characterized by the clonal proliferation of malignant plasma cells within the bone marrow, representing the second most common hematological malignancy worldwide [1,2]. While modern therapeutic strategies have led to significantly prolonged survival, the clinical course of MM remains defined by eventual relapses, with the disease becoming refractory to a substantial proportion of established agents [3,4]. Management of relapsed/refractory disease is a complex, risk-stratified procedure where treatment selection is based on several factors related to the disease, prior treatment, and the patient’s specific characteristics and preferences [5].

The treatment landscape for relapsed/refractory multiple myeloma (RRMM) has changed dramatically by the incorporation of B-cell maturation antigen (BCMA)-directed chimeric antigen receptor (CAR) T-cell therapies [6,7]. Idecabtagene vicleucel (ide-cel) was the first to demonstrate deep and durable responses in the pivotal KarMMa-3 trial that led to its approval, showing an overall response rate (ORR) of 71% and a median progression-free survival (PFS) of 13.3 months in patients treated with a median of 3 prior lines of therapy [8]. Notably, ide-cel demonstrated a 51% reduction in the risk of disease progression or death compared to standard-of-care (SOC) regimens (HR = 0.49, 95%CI: 0.38–0.65). Subsequently, ciltacabtagene autoleucel (cilta-cel) showed unprecedented efficacy in the CARTITUDE-4 study, with an ORR of 84.6% and a 12-month PFS rate of 75.9% in patients treated with a median of 2 prior lines of therapy [9]. In this earlier-line setting, treatment with cilta-cel resulted in a 74% reduced risk of disease progression or death (HR = 0.26, 95%CI: 0.18–0.38) compared to SOC, further establishing the potency of CAR T-cell intervention in RRMM.

Despite their regulatory approval, the widespread adoption of CAR T-cell therapy is often constrained by significant logistical and economic barriers, including high manufacturing costs, intensive resource requirements for administration, and the necessity of specialized certified infusion centers [10,11]. Moreover, a significant discrepancy remains between the highly selected populations of pivotal clinical trials and the heterogenous nature of real-world cohorts [12]. Trials often mandate stringent inclusion criteria, frequently excluding patients with significant comorbidities or impaired performance status. Consequently, the efficacy and toxicity profiles observed in controlled settings may not fully translate to unselected populations, where patients often present with higher disease burdens, frailty, and cytopenias. Despite that, given the aforementioned logistical barriers, few real-world studies have been published which are frequently limited by modest cohort sizes, restricting the generalizability of their findings. This highlights a critical need for large-scale real-world evidence to validate whether the superior outcomes of CAR T-cell therapy persist in clinical practice.

Using the TriNetX global health research network, we describe a large-scale, multi-institutional real-world experience of 1272 patients with RRMM treated with either cilta-cel or ide-cel. The main objective of this analysis is to provide a comprehensive description of overall survival and safety outcomes, also focusing on clinically relevant subgroups, for both approved CAR T-cell regimens.

## 2. Materials and Methods

### 2.1. Study Design

This retrospective, observational cohort study utilized real-world data from the TriNetX global health network (https://trinetx.com/, accessed on 25 February 2026), a federated database providing access to de-identified electronic health records (EHRs) from over 100 healthcare organizations (HCOs) worldwide, primarily large academic medical centers and specialized clinics. Adult patients with RRMM were retrospectively enrolled in this study. Patients were then stratified in separate cohorts, according to the CAR T-cell construct used (cilta-cel or ide-cel).

### 2.2. Population-Defining ICD-10 Codes

Patients were identified using the code C90.0 (Multiple Myeloma) and relapsed/refractory stage was confirmed via the built-in “treatment pathways” tool. The RxNorm codes 2594775 and 2536430 were utilized to further identify patients treated with cilta-cel or ide-cel, respectively.

### 2.3. Index Event and Date

The index event, defined with the TriNetX Analytics tools (https://trinetx.com/data-sets-analytics/, accessed on 25 February 2026), is the point in time when each patient in the cohort enters the analysis. The index date for each patient was defined as the first recorded administration of cilta-cel or ide-cel.

### 2.4. Outcomes

The primary outcome was Overall Survival (OS), defined as the time from the index date to death from any cause. Patients without a recorded death were censored at the date of their last interaction with the healthcare system. Progression-free Survival (PFS), defined as the time from the index date to death from any cause or disease progression, complimented OS as an efficacy outcome. Safety outcomes studied included key adverse events (AEs) of interest for CAR T-cells: cytokine release syndrome (CRS, any-grade or grade ≥ 3), immune effector cell-associated neurotoxicity syndrome (ICANS, any-grade or grade ≥ 3), infections of any type (any-grade), non-familial hypogammaglobulinemia (any-grade), neutropenia (grade ≥ 3), anemia (grade ≥ 3) and thrombocytopenia (grade ≥ 3). Specifically for patients with moderate-to-severe renal impairment (eGFR < 45 mL/min/1.73 m^2^ by CKD-EPI at the time of infusion), acute kidney injury was also assessed. Database codes that define the outcomes studied are listed in the Supplementary Material (Supplementary Table S1).

### 2.5. Statistical Analysis

Baseline characteristics were summarized using medians with interquartile ranges (IQR) or ranges for continuous variables, and frequencies with percentages for categorical variables. All AEs of interest are reported as crude numbers and/or percentages among evaluable patients. Kaplan–Meier curves were used to estimate survival and time-to-event probabilities. Univariate and multivariable Cox proportional hazards regression models were constructed to identify independent predictors of mortality. Hazard Ratios (HRs) and 95% Confidence Intervals (CIs) were calculated. All statistical analyses were performed within the TriNetX Analytics platform. In case of missing data, the TriNetX Analytics platform provides results based on patients with available data. Subgroup analyses were prespecified and included the stratification and subsequent analysis of the overall cohorts by age (< or ≥65 years), ISS staging, renal function (by CKD-EPI) and prior exposure to anti-CD38 monoclonal antibodies. Plots were recreated in R-Studio by utilizing extracted aggregate data. Statistical significance was defined as a two-sided *p*-value < 0.05. Given the exploratory nature of this real-world analysis and the limited number of pre-defined clinical subgroups evaluated, no formal adjustments for multiple comparisons were applied.

## 3. Results

A total of 697- and 575-MM patients were treated with cilta-cel and ide-cel, respectively. The median follow-up for the two cohorts was 237 (IQR 380) and 571 (IQR 599) days, respectively. At treatment initiation, the median age of patients was 65 (range 24–84) and 67 (range 30–89) years, respectively. There was a slight male predominance in both cohorts (56.2% and 59.8%), most of the patients were white (72.0% and 73.9%) and the next most frequent race was Black or African American (16.2% and 16.7% respectively). All of the patients were previously treated with at least a proteasome inhibitor and/or an immunomodulatory drug, while only the 1.0% of patient in the cilta-cel arm and the 3.0% in the ide-cel arm were previously exposed to anti-BCMA agents. Table 1 provides an overview of the baseline characteristics of both cohorts.

### 3.1. Survival Outcomes: Cilta-Cel

**Figure 1:**
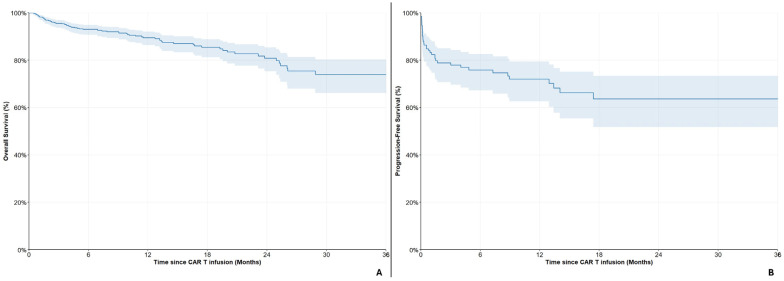
Overall survival (**A**) and Progression-free survival (**B**) in the cilta-cel cohort. Shaded area represents 95% CI.

Out of the 697 patients treated with Cilta-cel, a total of 74 (10.6%) deaths were recorded during follow-up. The median OS was not reached; 6- and 12-month OS rates were 93.1% (95%CI: 90.7–94.9%) and 89.6% (95%CI: 86.4–92.0%), respectively (Figure 1A). Among 133 evaluable patients, 37 (27.8%) patients exhibited disease progression or death. The median PFS was not reached; 6- and 12-month survival rates were 75.9% (95%CI: 67.3–82.6%) and 72.0% (95%CI: 62.6–79.5%), respectively (Figure 1B).

#### Subgroup Analyses in the Cilta-Cel Cohort

**Figure 2:**
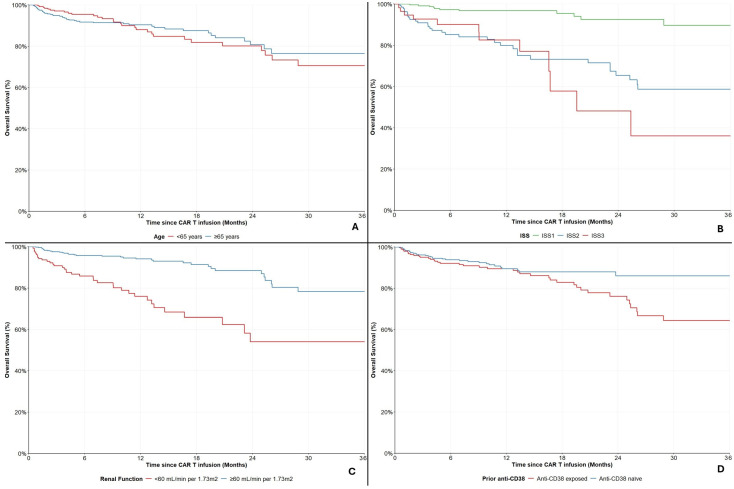
Overall survival in the cilta-cel cohort, stratified by (**A**) age, (**B**) ISS, (**C**) renal function and (**D**) prior anti-CD38 exposure.

Out of the 697 patients treated with cilta-cel, 415 (59.5%) were ≥65 years old. The 6- and 12-month OS rates between those <65 years old were 95.5% (95%CI: 91.7–97.5%) and 88.0% (95%CI: 81.5–92.4%) and between those ≥65 years old 91.7% (95%CI: 88.4–94.2%) and 90.4% (95%CI: 86.6–93.1%), respectively (Figure 2A). No differences were noted between the two groups (HR = 0.97, 95%CI: 0.61–1.55, *p* = 0.900).

Laboratory markers for ISS evaluation were available for 475 patients (68.2%). Among them, 274 (57.7%) were categorized as ISS-1, 142 (29.9%) as ISS-2 and 59 (12.4%) as ISS-3. The 6- and 12-month OS rates for ISS-1 patients were 97.3% (95%CI: 94.1–98.8%) and 96.7% (95%CI: 93.3–98.5%), for ISS-2 patients 85.3% (95%CI: 77.7–90.4%) and 80.0% (95%CI: 71.0–86.4%) and for ISS-3 90.0% (95%CI: 77.5–95.8%) and 82.5% (95%CI: 65.4–91.7%), respectively (Figure 2B).

A total of 160 patients (23.0%) had impaired renal function (<60 mL/min per 1.73 m^2^). The 6- and 12-month OS rates for these individuals were 85.8% (95%CI: 78.8–90.7%) and 75.9% (95%CI: 66.5–83.2%) compared to 95.8% (95%CI: 93.4–97.4%) and 94.1% (95%CI: 91.1–96.2%) for those with normal renal function (Figure 2C). Impaired renal function seemed to be a factor of adverse prognosis in patients treated with cilta-cel (HR = 3.66, 95%CI: 2.30–5.83, *p* < 0.001).

A total of 256 (36.7%) patients had been exposed to regimens including anti-CD38 monoclonal antibodies, prior to treatment with cilta-cel. The 6- and 12-month OS rates for those were 92.1% (95%CI: 87.7–95.0%) and 89.5% (95%CI: 84.4–93.0%), compared to 93.9% (95%CI: 90.8–95.9%) and 89.5% (95%CI: 85.0–92.8%) for those who were anti-CD38 monoclonal antibodies naïve, respectively (Figure 2D). A non-significant trend of worse prognosis was observed for patients who were exposed to treatment with anti-CD38 antibodies in lines prior to cilta-cel infusion (HR = 1.49, 95% CI: 0.92–2.43, *p* = 0.103).

### 3.2. Survival Outcomes: Ide-Cel

**Figure 3:**
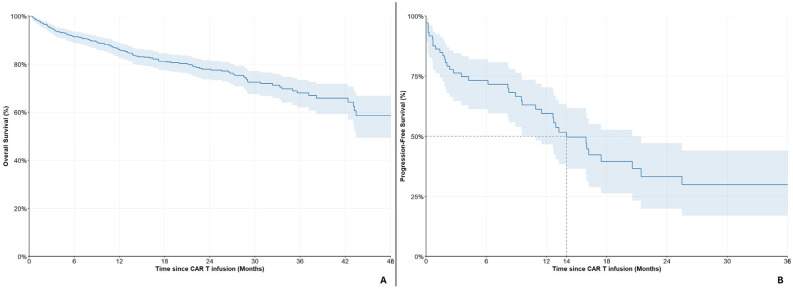
Overall survival (**A**) and Progression-free survival (**B**) in the ide-cel cohort. Shaded area represents 95% CI.

Out of 575 patients treated with Ide-cel, a total of 127 (22.1%) deaths were recorded during follow-up; the median OS was 5.1 years. The 12-month, 18-month and 24-month OS rates were 86.0% (95%CI: 82.7–88.8%), 81.3% (95%CI: 77.4–84.5%) and 78.0% (95%CI: 73.7–81.6%), respectively (Figure 3A). Only 73 patients were evaluable for PFS, out of which 39 (53.4%) patients exhibited disease progression or death. The median PFS was 14 months and the 6-month and 12-month PFS rates were 73.3% (95%CI: 61.3–82.1%) and 59.6% (95%CI: 46.5–70.4%), respectively (Figure 3B).

#### Subgroup Analyses in the Ide-Cel Cohort

**Figure 4:**
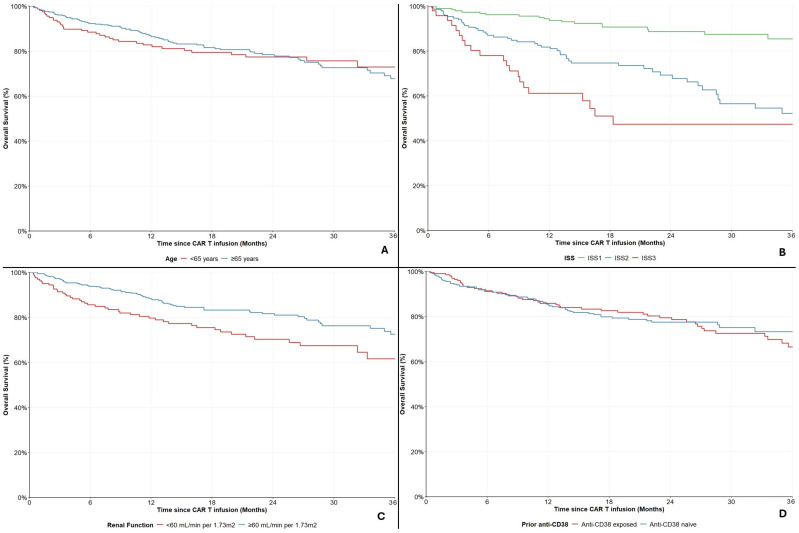
Overall survival in the ide-cel cohort, stratified by (**A**) age, (**B**) ISS, (**C**) renal function and (**D**) prior anti-CD38 exposure.

Out of 575 patients treated with ide-cel, 391 (68.0%) were ≥65 years old. The 12- and 24-month OS rates among them were 86.5% (95%CI: 82.3–89.7%) and 78.5% (95%CI: 73.2–82.9%), compared to those younger than 65 years at 82.0% (95%CI: 74.8–87.3%) and 77.4% (95%CI: 69.4–83.6%), respectively (Figure 4A). No differences were noted between the two groups (HR = 1.01, 95%CI: 0.68–1.50, *p* = 0.966).

Laboratory markers for ISS evaluation were available for 394 patients (68.5%). Among them, 192 (48.7%) were ISS-1, 154 (39.1%) were ISS-2 and 48 (12.2%) were ISS-3. The 12-month OS rate for ISS-1 patients was 93.6% (95%CI: 88.9–96.5%), for ISS-2 81.8% (95%CI: 75.5–87.1%) and for ISS-3 61.2% (95%CI: 45.1–73.9%), respectively (Figure 4B).

A total of 164 patients (28.5%) had impaired renal function (<60 mL/min per 1.73 m^2^). The 12- and 24-month OS rates for these individuals were 79.7% (95%CI: 72.3–85.3%) and 70.3% (95%CI: 61.6–77.5%), compared to 88.1% (95%CI: 84.2–91.2%) and 81.7% (95%CI: 76.7–85.7%) for those with normal renal function, respectively (Figure 4C). Impaired renal function seemed to be a factor of adverse prognosis in patients treated with ide-cel (HR = 1.73, 95%CI: 1.20–2.51, *p* = 0.003).

A total of 207 (36.0%) patients had been exposed to regimens with anti-CD38 monoclonal antibodies, prior to treatment with ide-cel. The 12- and 24-month OS rates for those were 85.9% (95%CI: 80.1–90.0%) and 79.5% (95%CI: 72.5–84.8%), compared to 85.1% (95%CI: 80.5–88.7%) and 77.5% (95%CI: 71.8–82.2%) for those who were anti-CD38 naïve, respectively (Figure 4D). No differences were noted between these two groups (HR = 1.06, 95% CI: 0.73–1.53, *p* = 0.770).

### 3.3. Safety

#### 3.3.1. Systemic and Neurological Toxicities

Any-grade Cytokine Release Syndrome (CRS) was reported in 45.9% (192/418) of patients in the cilta-cel cohort and 41.8% (99/237) in the ide-cel cohort. Immune Effector Cell-Associated Neurotoxicity Syndrome (ICANS) of any-grade occurred in 15.4% (95/618) and 11.8% (54/456) of patients, respectively. Severe neurological toxicity (grade ≥ 3 ICANS) was rare, affecting only 1.8% (11/618) of the cilta-cel cohort and 2.9% (13/456) of the ide-cel cohort.

#### 3.3.2. Infections and Hypogammaglobulinemia

Infections occurred in 28.5% (80/281) of patients treated with cilta-cel compared to 40.1% (79/197) of those treated with ide-cel. Furthermore, hypogammaglobulinemia was highly prevalent in both groups, recorded in 41.1% (180/438) of the cilta-cel cohort and 43.1% (160/371) of the ide-cel cohort. The 54.1% (377/697) of patients treated with cilta-cel initiated immunoglobulin G supplementation post CAR T infusion, compared to the 51.8% (298/575) among those treated with ide-cel.

#### 3.3.3. Serious Hematologic Toxicities

**Figure 5:**
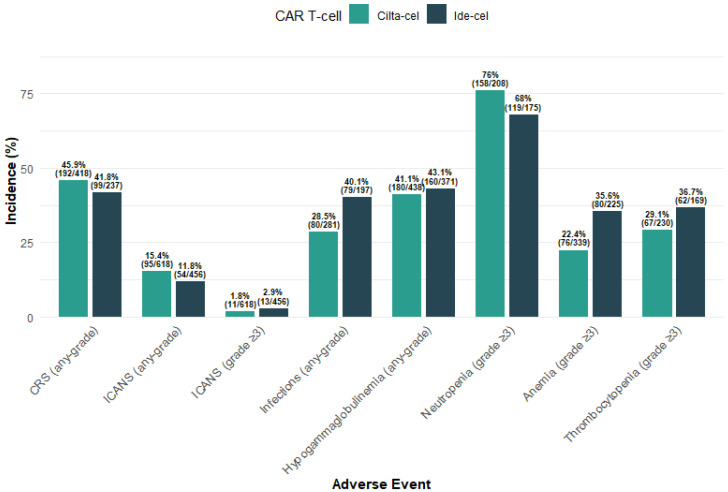
Safety Profile of Cilta-cel and Ide-cel.

Grade ≥ 3 hematologic toxicities were frequent. Grade ≥ 3 neutropenia was observed in 76.0% (158/208) of cilta-cel patients and 68.0% (119/175) of ide-cel patients. Grade ≥ 3 anemia occurred in 22.4% (76/339) and 35.6% (80/225) of patients, respectively. Finally, grade ≥ 3 thrombocytopenia was noted in 29.1% (67/230) and 36.7% (62/169) of the two cohorts (Figure 5).

### 3.4. Exploratory Sub-Analysis in Patients with Moderate-to-Severe Renal Impairment

A sub-population of patients in these real-world cohorts received CAR T-cell therapy while having moderate-to-renal impairment. Specifically, 10.5% (*n* = 73/697) of the cilta-cel cohort and 15.1% (*n* = 87/575) of the ide-cel cohort had an eGFR < 45 mL/min/1.73 m^2^ at the time of infusion. Within these subgroups, severe renal impairment (eGFR < 30 mL/min/1.73 m^2^) was present in 4.9% (*n* = 34) of patients treated with cilta-cel and 8.5% (*n* = 49) of those treated with ide-cel.

The 12-month OS rates were 70.3% (95%CI: 53.4–82.0%) and 72.3% (95%CI: 60.8–80.7%), respectively. Among evaluable patients, the incidence of any-grade or serious CRS and ICANS was rare (<2%). In the cilta-cel cohort, serious anemia and thrombocytopenia events were also limited (<1.5%), while grade ≥ 3 neutropenia and hypogammaglobulinemia occurred in 80.8% and 28.9% of evaluable patients, respectively. In the ide-cel cohort, in the evaluable population, grade ≥ 3 anemia occurred in 56.5%, grade ≥ 3 neutropenia in 75.0%, grade ≥ 3 thrombocytopenia in 55% and hypogammaglobulinemia in 47.2%. Notably, while acute kidney injury was rare in the cilta-cel cohort (<1.5%), it was observed in 40.6% of evaluable patients in the ide-cel cohort.

## 4. Discussion

Authors This large-scale, multi-institutional analysis utilizing the TriNetX health research network demonstrates that approved BCMA-directed CAR T-cell therapies, cilta-cel and ide-cel, are both feasible and effective in a diverse, real-world population of RRMM patients. Crucially, our findings suggest that the clinical benefit of these agents is largely preserved in routine practice, despite the inclusion of unselected patients who would have potentially been ineligible for pivotal trials.

Our analysis reveals real-world OS rates that align favorably with those reported in the pivotal clinical trials that led to regulatory approvals, despite the inclusion of an unselected patient population. In the cilta-cel cohort, we observed a 12-month OS rate of 89.6%, which is consistent with the CARTITUDE-1 and CARTITUDE-4 results, where the 12-month OS was 89.0% and 84.1%, respectively [8,13]. Similarly, our ide-cel cohort’s 12- and 24-month OS rates of 86.0% and 78.0% compare favorably to the KarMMa study, which reported a 12-month OS of 78% in the triple-class exposed setting, and the updated KarMMa-3 analysis showing 12- and 24-month OS rates of approximately 75% and 65%, respectively [9,14,15]. While PFS data in the present study were limited by the availability of evaluable structured records, the 12-month PFS rates in evaluable subsets (72.0% for cilta-cel and 59.6% for ide-cel) were comparable to those of the randomized CARTITUDE-4 (cilta-cel, 75.9%) and KarMMa-3 (ide-cel, 55.0%) trials [8,9].

The safety profile observed in our real-world cohorts generally reflects the systemic and hematologic toxicities identified in clinical trials, though with notable variations in reporting frequency. The incidence of any-grade CRS in our study (45.9% for cilta-cel and 41.8% for ide-cel) was lower than the rates reported in CARTITUDE-1 (95%) and KarMMa (84%) [9,13]. This difference likely arises from the retrospective nature of electronic health record data and variations in real-world institutional coding versus the proactive, daily monitoring required in trial protocols [16]. However, our findings confirm that severe neurological toxicity (grade ≥ 3 ICANS) remains rare in everyday clinical practice (<3%), mirroring the safety data from the randomized KarMMa-3 and CARTITUDE-4 studies (0.1% and 3%) [8,9]. High-grade hematologic toxicities were a dominant feature of the real-world experience, with grade ≥ 3 neutropenia occurring in 76.0% (cilta-cel) and 68.0% (ide-cel), slightly lower but comparable to the near-universal rates observed in trial populations (89.9% in CARTITUDE-4 and 78.0% in KarMMa-3) [8,9]. Importantly, our data highlight a notable prevalence of infections (28.5% for cilta-cel and 40.1% for ide-cel) and hypogammaglobulinemia (41.1% for cilta-cel and 43.1% for ide-cel). The latter aligns with the findings of CARTITUDE-1 (42.3%) and CARTITUDE-4 (45.2%) trials [8,13], suggesting that BCMA-directed therapies necessitate vigilant, long-term monitoring for secondary immunodeficiencies outside of the controlled trial environment. Vaccination, infection prophylaxis and supplementation with intravenous or subcutaneous gamma globulins should be integrated to minimize infection rate [17,18].

Our findings are in accordance with two recent multicenter real-world studies that demonstrate similar results both in patient demographics and clinical outcomes. In an analysis of standard-of-care cilta-cel administration in 236 patients, Sidana et al. observed that 54% of would not have met the eligibility criteria for the CARTITUDE-1 trial, primarily due to comorbidities, organ dysfunction, or prior BCMA-targeted therapy [19]. Despite this high rate of trial ineligibility, they reported a 12-month OS estimate of 82%, which aligns with the favorable survival observed in our unselected cohort and the estimates of the published clinical trials. Similarly, in another real-world cohort (*n* = 223), Bhutani et al. noted that 41% of minority patients and 24% of white patients in their study would have been ineligible for the pivotal KarMMa and CARTITUDE trials [20]. In this study, the majority of patients were treated with ide-cel and the observed median PFS of 15.8 and 14.1 months in white and minority patients, respectively, aligns with the observations in our ide-cel cohort (median PFS 14.0 months). Furthermore, the 12- and 18-month OS rates in the range of 80–85% and 70–75%, respectively, are consistent with our findings in the ide-cel cohort.

The safety profile in our study also reflects these recent real-world reports. While our observed rates of any-grade CRS (41.8–45.9%) were numerically lower than those reported by Sidana et al. (75%) and Bhutani et al. (80–87%), all three studies consistently demonstrate that severe neurological toxicity (grade ≥ 3 ICANS) remains rare in clinical practice, typically occurring in less than 4% of patients [19,20]. Finally, the significant burden of infections identified in our cohorts (28.5–40.1%) is supported by Sidana et al., who reported infections in 47% of the patients with nearly half of those being classified as severe [19].

Furthermore, our analysis identifies baseline renal dysfunction (eGFR < 60 mL/min/1.73 m^2^) as a potential factor of adverse prognosis, with a significantly higher prevalence in clinical practice than in pivotal trial populations. While our cohorts included a substantial proportion of patients with renal dysfunction (23.0% of cilta-cel and 28.5% of ide-cel patients), pivotal trials largely excluded this demographic through strict eligibility criteria. For instance, the CARTITUDE-4 trial included only 27 patients (13.0%) with renal dysfunction of any grade. Our findings of significantly inferior OS in patients with eGFR < 60 (cilta-cel HR: 3.66; ide-cel HR: 1.73) underscore a notable “real-world” challenge that is less apparent in trial settings. Additionally, while the CARTITUDE-4 and KarMMa-3 trials utilized eGFR cutoffs of 40 and 45 mL/min/1.73 m^2^, respectively, we identified a substantial subset (10.5% of cilta-cel; 15.1% of ide-cel) treated below these thresholds. Despite moderate-to-severe baseline renal dysfunction, these patients achieved favorable 12-month OS rates with no disproportionate CRS/ICANS signals. However, high rates of grade ≥ 3 cytopenias and a distinct incidence of acute kidney injury in the ide-cel cohort (40.6%) underscore the need for vigilant monitoring in these high-risk phenotypes, where treatment channeling often favors the product with the most immediate availability. While these observations must be interpreted with caution due to the limited number of evaluable patients in these subgroups, they are further supported by emerging evidence, such as a recent case series demonstrating the safety and feasibility of cilta-cel even in dialysis-dependent RRMM patients [21].

These results contrast with findings from other multicenter real-world studies, which suggest that while renal impairment significantly increases toxicity, it may not inherently compromise efficacy or survival outcomes. In a retrospective study of 223 patients, those with a creatinine clearance (CrCl) < 45 mL/min achieved comparable overall response rates and survival compared to those with normal renal function, despite elevated rates of ICANS and infections [22]. Similarly, Sidana et al. found in a cohort of 75 ide-cel patients that baseline renal function-with CrCl < 50 mL/min as a cutoff-did not significantly impact PFS or OS, despite RI patients experiencing higher rates of grade ≥ 3 cytopenias [23]. The survival discrepancy noted in our study may reflect the broader TriNetX population’s heterogeneity, as both Atrash et al. and Sidana et al. emphasized that while CAR T-cell therapy is feasible in RI, these patients often present with higher inflammatory markers and a more aggressive baseline phenotype that necessitates vigilant clinical monitoring [24].

Notably, our analysis also suggests that prior exposure to anti-CD38 monoclonal antibodies does not significantly compromise the efficacy of subsequent BCMA-directed CAR T-cell therapy. We observed comparable 12-month OS rates between anti-CD38 exposed and naïve patients across both the cilta-cel (HR = 1.49, *p* = 0.103) and ide-cel cohorts (HR = 1.06, *p* = 0.770). This finding is particularly relevant given the increasing use of anti-CD38 agents in the frontline or asymptomatic setting [25,26,27,28,29] and as a 2nd line treatment in combination with teclistamab [30], and it aligns with observations from the CARTITUDE and KarMMa trials where patients achieved robust responses regardless of prior CD38-targeted exposure. However, these observations must be interpreted with caution. Given the retrospective nature of our data, this specific comparison is inherently susceptible to unmeasured confounders, such as the exact timing of the prior anti-CD38 therapy, the duration of exposure, and the patient’s specific refractoriness status. Consequently, while these data support the potential role of CAR T-cell therapy as a highly effective salvage strategy for patients who have progressed on current standard-of-care anti-CD38 regimens [31,32], prospective validation is required to definitively confirm these findings while accounting for these clinical variables.

The primary strength of this study lies in its sample size (*n* = 1272), which represents one of the largest real-world cohorts for BCMA-directed CAR T-cells to date. Furthermore, our cohort achieved a high degree of racial diversity, with 16% Black/African American representation, a population underrepresented in the pivotal CARTITUDE and KarMMa trials. By utilizing a global health research network, this study—as one of the few real-world analyses to date—provides valuable insights into the safety and efficacy profile of BCMA-directed CAR T-cell therapies in routine clinical practice.

Despite its strengths, the study also carries several limitations inherent to the use of electronic health record (EHR) data. First, the retrospective nature of the TriNetX database depends on the accuracy of ICD-10 and RxNorm coding, which may lead to an underestimation of early-grade toxicities like grade 1–2 CRS compared to the active monitoring applied in clinical trials. Second, there is a notable “non-availability” of laboratory and cytogenetic data; for example, R-ISS staging and other high-risk cytogenetic markers were not available for the entire cohort. Additionally, PFS was only evaluable in a small subset of patients due to the lack of standardized clinical progression proxies (e.g., IMWG response criteria) within the TriNetX platform, which instead relies on specific coding. This missingness introduces potential selection bias as patients with fully evaluable PFS trajectories represent only a specific subpopulation, limiting the generalizability of the disease control observed to the broader real-world cohort. Third, while we identified renal impairment as a factor of adverse prognosis, the data available cannot discriminate whether this was due to increased treatment-related toxicity or more aggressive underlying disease biology. Moreover, in this context, the analytical constraints of the platform precluded comprehensive multivariable adjustments for confounders such as cumulative prior therapies overall disease burden or staging and high-risk cytogenetics, thus limiting our ability to definitively establish the prognostic importance of our subgroup analyses. Fourth, detailed grading was not universally available in TriNetX for all of the AEs studied. Furthermore, because outcome classification depends entirely on the presence of structured clinical records, the denominators for specific adverse events fluctuate based on the number of patients with evaluable data for that specific toxicity. Fifth, the heterogeneity of data sources within the TriNetX network reflects a broad spectrum of real-world clinical practices, but it also introduces variability in how outcomes or AEs are monitored and coded. Finally, while this study provides a large-scale look at both cilta-cel and ide-cel, the lack of a randomized comparison and the presence of baseline imbalances between the cohorts mean these results should be viewed as descriptive observations rather than a direct comparative assessment of the two products. Consequently, these results are intended to complement, rather than substitute, the high-level evidence provided by prospective studies or controlled clinical trials.

## 5. Conclusions

In conclusion, this large-scale, observational, retrospective analysis of 1272 patients suggests that the approved BCMA-directed CAR T-cell therapies, cilta-cel and ide-cel, are associated with robust efficacy and a manageable safety profile in routine clinical practice. Furthermore, the comparable outcomes observed regardless of prior anti-CD38 exposure highlight CAR T-cell therapy as a highly effective treatment option even in more heavily pretreated patients. However, given the inherent limitations of real-world observational data, these findings should be interpreted as associative rather than causal. Ultimately, these real-world data support the continued integration of CAR T-cell therapies into standard care, while emphasizing the critical need for vigilant monitoring of early- and late-onset toxicities.

## Figures and Tables

**Table 1:** Baseline population characteristics.

	Cilta-Cel (*n* = 697)	Ide-Cel (*n* = 575)
Age (years)—median (range)	65 (range 24–84)	67 (range 30–89)
Males—*n* (%)	392 (56.2)	344 (59.8)
Race—*n* (%)		
White	502 (72.0)	425 (73.9)
Black or African American	113 (16.2)	96 (16.7)
Asian	15 (2.2)	10 (1.7)
Other	67 (9.6)	44 (7.7)
Hispanic or Latino—*n* (%)	30 (4.3)	29 (5.0)
Extramedullary plasmacytoma—*n* (%)	10 (1.4)	15 (2.3)
ISS ^a^—*n* (%)		
1	274 (57.7)	192 (48.7)
2	142 (29.9)	154 (39.1)
3	59 (12.4)	48 (12.2)
ECOG PS—median (range)	1 (0–3)	1 (0–3)
eGFR ^b^—median (range)	85.8 (30.4)	82.8 (36.5)
Renal dysfunction ^c^—*n* (%)	160 (23.0)	164 (28.5)
Previous LoT ^a^—median (range)	3 (1–6)	4 (1–6)
PI and/or IMiD exposure—*n* (%)	697 (100.0)	575 (100.0)
Anti-CD38 exposure—*n* (%)	256 (36.7)	207 (36.0)
Daratumumab	246 (35.3)	196 (34.1)
Isatuximab	10 (1.4)	11 (1.9)
Anti-BCMA exposure—*n* (%)	7 (1.0)	15 (2.6)

^a^ Among patients with available laboratory data, ^b^ eGFR by CKD-EPI, ^c^ Renal dysfunction defined as CrCl < 60 mL/min per 1.73 m^2^.

## Data Availability

The data analyzed was accessed via the TriNetX global research network platform and is available there.

## Supplementary Materials

The following supporting information can be downloaded at: https://www.mdpi.com/article/10.3390/cancers18061013/s1, Table S1. Codes utilized in the TriNetX database in order to define the outcomes studied.
