# Supplementary material for: Real-World Experience with Approved CAR T-Cell Therapies Ciltacabtagene Autoleucel and Idecabtagene Vicleucel in 1272 Relapsed/Refractory Multiple Myeloma Patients
Source: Cancers (Basel). 2026 Mar 20;18(6):1013. doi: 10.3390/cancers18061013 (PMC13024817; doi:10.3390/cancers18061013)
Supplement: Supplementary file 1 [file cancers-18-01013-s001.zip › cancers-4199232-supplementary.pdf]

Real-World Experience with Approved CAR T-Cell Therapies Ciltacabtagene Autoleucel and Idecabtagene Vicleucel in 1272 Relapsed/Refractory Multiple Myeloma Patients

Supplementary Material

Supplementary Table S1. Codes utilized in the TriNetX database in order to define the outcomes studied

**Outcome**	**Code**
Relapse	C90.02
CRS, any-grade	D89.83
ICANS, any-grade	G92.0
CRS, grade ≥3	D89.833-5
ICANS, grade ≥3	G92.03-5
Anemia, grade ≥3	Hemoglobin <8.0 g/dL (9014)
Neutropenia, grade ≥3	Neutrophils <1000/uL (9018)
Thrombocytopenia, grade ≥3	Platelets <50000/uL (9020)
Infections, any-grade	A00-B99
Hypogammaglobulinemia, any-grade	D80.1
Acute kidney injury	N17
